# Evolution of Natural Product Scaffolds as Potential Proteasome Inhibitors in Developing Cancer Therapeutics

**DOI:** 10.3390/metabo13040509

**Published:** 2023-03-31

**Authors:** Reyaz Hassan Mir, Prince Ahad Mir, Jasreen Uppal, Apporva Chawla, Mitesh Patel, Fevzi Bardakci, Mohd Adnan, Roohi Mohi-ud-din

**Affiliations:** 1 Pharmaceutical Chemistry Division, Department of Pharmaceutical Sciences, University of Kashmir, Hazratbal, Srinagar 190006, Jammu and Kashmir, India; 2 Khalsa College of Pharmacy, G.T. Road, Amritsar 143001, Punjab, India; 3 Department of Biotechnology, Parul Institute of Applied Sciences and Centre of Research for Development, Parul University, Vadodara 391760, Gujarat, India; 4 Department of Biology, College of Science, University of Ha’il, Ha’il P.O. Box 2440, Saudi Arabia; 5 Department of General Medicine, Sher-I-Kashmir Institute of Medical Sciences (SKIMS), Srinagar 190001, Jammu and Kashmir, India

**Keywords:** ubiquitin-proteasome pathway, proteasome inhibitor, cancer, natural compounds, drug discovery, protein degradation, secondary metabolites, clinical trial

## Abstract

Homeostasis between protein synthesis and degradation is a critical biological function involving a lot of precise and intricate regulatory systems. The ubiquitin-proteasome pathway (UPP) is a large, multi-protease complex that degrades most intracellular proteins and accounts for about 80% of cellular protein degradation. The proteasome, a massive multi-catalytic proteinase complex that plays a substantial role in protein processing, has been shown to have a wide range of catalytic activity and is at the center of this eukaryotic protein breakdown mechanism. As cancer cells overexpress proteins that induce cell proliferation, while blocking cell death pathways, UPP inhibition has been used as an anticancer therapy to change the balance between protein production and degradation towards cell death. Natural products have a long history of being used to prevent and treat various illnesses. Modern research has shown that the pharmacological actions of several natural products are involved in the engagement of UPP. Over the past few years, numerous natural compounds have been found that target the UPP pathway. These molecules could lead to the clinical development of novel and potent anticancer medications to combat the onslaught of adverse effects and resistance mechanisms caused by already approved proteasome inhibitors. In this review, we report the importance of UPP in anticancer therapy and the regulatory effects of diverse natural metabolites, their semi-synthetic analogs, and SAR studies on proteasome components, which may aid in discovering a new proteasome regulator for drug development and clinical applications.

## 1. Introduction

Cancer is the uncontrollable growth of cells that have undergone somatic mutations which endow them with unusual characteristics that are responsible for their abnormal behavior. Resistance to cell death, overexpression of a proliferative signal transduction pathway, abrogation of growth suppressing signals, and the ability to metastasize and undergo angiogenesis are some of the traits associated with the cancerous cells [1]. Cancer is caused by a multitude of factors that are complex and poorly understood. Several external influences (radiation, a habit of chewing or smoking of tobacco, infectious organisms, etc.) and certain internal characteristics (immune conditions, inherited and random mutations, imbalance of hormones, etc.) lead to the development of disease [2]. 

### Ubiquitin-Proteasome Pathway (UPS)

**Figure 1:**
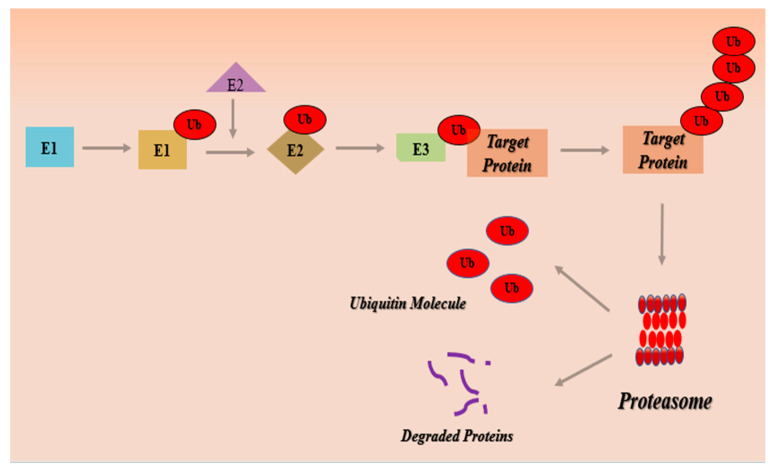
Ubiquitin-proteasome pathway.

The proteins are complex molecules made up of amino acids and serve as basic fundamental building blocks of all living species. Protein homeostasis is a dynamic equilibrium between protein synthesis and protein degradation that is essential for the maintenance of healthy cell activities and the prevention of diseases [3]. The ubiquitin-proteasome pathway (Figure 1) maintains cellular protein homeostasis and mediates the degradation of various regulatory proteins involved in biological processes—notably, those proteins that regulate cell survival, signal transduction, and DNA repair [4]. The proteasome is a large multi-protease complex that degrades most intracellular proteins and accounts for about 80% of cellular protein degradation [5,6]. Degradation of misfolded and potentially harmful oxidized proteins, and the controlled proteolytic breakdown of regulatory proteins, including those that are engaged in apoptosis and cell cycle progression, are predominantly regulated by the ubiquitin-proteasome pathway (UPP) [7]. Proteins intended for proteolytic degradation are covalently linked with short polypeptide ubiquitin chains through a process of ubiquitylation [8]. Proteins that conjugate with the polyubiquitin chain are subsequently broken down into small peptides by the proteasome, and ubiquitin is liberated and recycled. Therefore, the ubiquitin-conjugating system and 26S proteasome work synergistically to drive this proteolytic activity [9,10]. 

The 20S proteasome comprises a 20S proteolytic core and a 19S regulatory protein. The core protein is cylindrical and has two identical α-subunit rings and two identical β-subunit rings. The 19S regulatory protein serves as a lid for the 20S barrel [11]. The 20S proteasome is a huge 700 kDa complex, where two outer α rings and two inner β rings are layered on top of one another. The constitutive active sites are β1, β2, and β5, which are responsible for caspase-like, trypsin-like, and chymotryptic activity, respectively [12]. To be degraded, proteins must be polyubiquitinated. The 19S regulatory protein is responsible for binding and recognizing the ubiquitinated proteins and directing them to the 20S core, where proteolytic cleavage is mediated by three β-subunits: β1, β2, and β5 [11]. The ubiquitin-proteasomal degradation cascade comprises ubiquitin, a three-enzyme ubiquitination complex, an intercellular protein target, and the proteasome, which serves as a degradation unit [13]. The ligation of ubiquitin to protein substrate is accomplished by stepwise processes involving enzymes such as ubiquitin-activating enzymes (E1), ubiquitin-conjugating enzyme (E2), and ubiquitin ligase (E3) [14]. E1 activates ubiquitin with the utilization of ATP. The E2 enzyme is responsible for receiving ubiquitin molecules from E1 and transporting them to E3. The E3 enzyme conjugates ubiquitin molecules to the lysine residues on target proteins generating a polyubiquitin chain that is subsequently degraded by a 20S proteasome complex [1].

Abnormal proteasome-mediated proteolysis has been associated with various cancer cells. Proteasome activity is often higher in cancer cells than normal cells, and cancer cells are more vulnerable to the pro-apoptotic effect of proteasome inhibition than a normal cell. Therefore, inhibition of proteasomal activity might be an effective and novel strategy against various malignancies, thereby rendering the proteasome an appropriate target in cancer [15]. Cancer cells rely heavily on proteasome-mediated protein degradation. As a result, treatment strategies have been focused on promoting direct proteasome inhibition, and subsequently increasing intracellular protein accumulation. An overabundance of misfolded protein load within the cell causes increased endoplasmic reticulum stress and cellular death. Consequently, the pharmacological agents targeting the proteasome are being explored widely in a variety of human malignancies [16]. Proteasome inhibitors are currently being employed to treat multiple myeloma and mantle cell lymphoma [17].

## 2. Natural Product Scaffolds as Proteasome Inhibitors against Cancer

Natural products have unique chemical diversity, which results in diversity in their biological activities and drug-like properties. Physical chemistry has been able to recognize the high structural diversity of natural products over the past century. Their efficacy is related to the complexity of their well-organized three-dimensional chemical and steric properties, which offer many advantages in terms of efficiency and the selectivity of molecular targets. Artemisinin and its analogues are currently used extensively for the treatment of malaria, serving as a successful example of medication from natural products. [18,19,20,21,22]. Furthermore, they are less harmful than existing chemotherapy medications [23,24,25,26]. These qualities, together with their widespread availability around the world, indicate that natural products could be employed in combination treatments. Natural compounds can be combined with current anti-cancer therapies to boost their effectiveness and help overcome resistance to chemotherapy drugs. They can also be used as starting points for developing new drugs by creating modified prodrugs or synthetic analogs of their natural counterparts [27,28,29]. Thus, in the hunt for novel pharmacologically active substances, natural products from various sources, such as plants, animals, microorganisms, and marine environments, have thus played an important role in treating numerous diseases. Here, intriguing research on natural products as anti-cancer therapeutics is thoroughly examined, focusing on their molecular targets, treatment, and prevention potential.

### 2.1. Plant-Derived Proteasome Inhibitors

#### 2.1.1. Emodin

**Figure 2:**
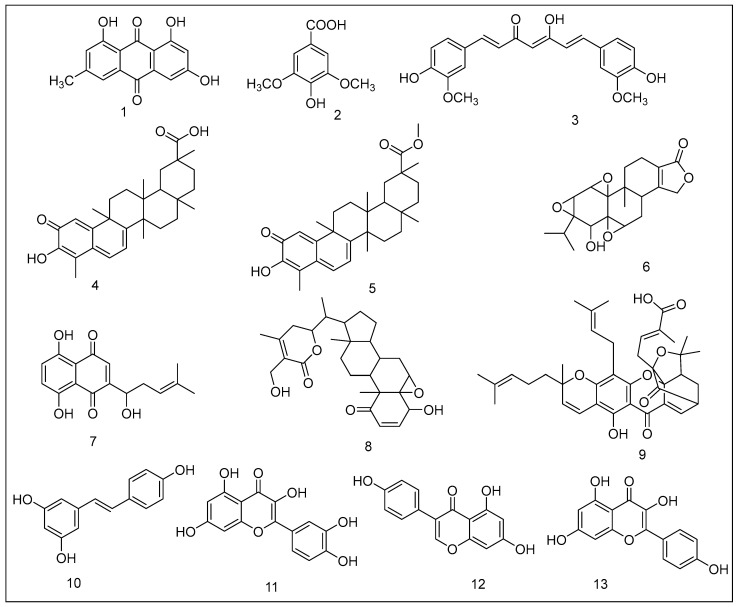
Plant-derived proteasome inhibitors: (**1**) emodin; (**2**) syringic acid; (**3**) curcumin; (**4**) celastrol; (**5**) pristimerin; (**6**) triptolide; (**7**) shikonin; (**8**) withaferin A; (**9**) gambogic acid; (**10**) resveratrol; (**11**) quercetin; (**12**) genistein; (**13**) kaempferol.

*Aloe vera’s* major active component, emodin (Figure 2), is a natural anthraquinone that exhibits anti-cancer and anti-inflammatory activity [30,31]. Emodin suppresses the cell cycle, stimulates the production of HIF-1α (hypoxia-inducible factor 1α), and prevents the production of the latter by addition reactions between carcinogenic agents and DNA [32]. Its resemblance to tricyclic anthracyclines, such as doxorubicin, appears to be an intriguing benchmark in the quest for the structural and pharmacological associations with anticancer activity. The biggest drawback of emodin for probable use as a treatment is its significant in-vivo toxicity and low bioavailability. In the gut and liver, this molecule experiences profound glucuronidation [33,34].

Emodin is likewise a strong blocker of the human 26S proteasome. It suppresses chymotrypsin-like activity (IC_50_ = 1.22 mM), caspase-related action (IC_50_ = 0.24 mM), and trypsin-related action (IC_50_ = 20.85 mM), and enhances endogenous proteins’ ubiquitination in the cell. Emodin also enhances nucleophilic assault in active positions of the proteasome. Docking studies suggest that emodin restricts the caspase-like proteasomal action by creating H-bonds and hydrophobic interactions, whereas the suppression of chymotrypsin-related action originates mostly from creating hydrophobic interactions [35]. HSP (heat shock protein) might be intricated in another mechanism that controls the UPS pathway reaction. Many cancers have high levels of HSPs, which help foster tumor growth. HSPs protect cancerous cells from programmed cell death and promote cancer growth by stabilizing the active products of a mutant gene [36].

Emodin derivatives influence the UPS system indirectly by blocking the production of a complex between signaling proteins and Hsp90 (a chaperone protein), which are often carcinogenic. Although model studies do not rule out the probability that the analog binds to both Hsp90 and Her2/neu, it has been discovered that an azide derivative of methyl anthraquinone impairs Hsp90 and Her2/neu binding. As a result, Her2/neu is degraded by proteasomes, resulting in cell death [37]. By activating the dissociation of ERa and HSP90, aloe-emodin (1,8-dihydroxy-3-hydroxymethylanthraquinone) inhibits the proliferation of estrogen-dependent breast cancer cells. Importantly, instead of translocating to the nucleus and acting as a transcriptional stimulator, the released ERa protein is ubiquitinated [38].

#### 2.1.2. Syringic Acid Derivatives

Syringic acid is a phenolic compound present in olives, pumpkin, dates, honey, spices, grapes, acai palm, and other plants that inhibits the Nox/PTP-k/EGFR pathway and has a substantial anti-proliferative action on colon, breast, and skin cancer cells [39,40,41]. Its anti-mitogenic effect is linked to the concurrent inhibition of the binding of NFkB to DNA, proteasomal suppression (ChT-L, PGPH), angiogenesis suppression, and cell sensitization to basic anticancer drugs (vincristine (130-times), camptothecin (500-times), taxol (3134-times), 5FU (20,000-times), vinblastine (1000-times), and doxorubicin (210-times) compared to these drugs alone in vitro [39]. Semi-synthetic analogs of this acid have been synthesized, demonstrating substantial antagonistic efficacy against the binding sites of the 20S proteasome. The following syringic acid analogs exhibited significant mitogenic actions on human malignant melanoma cells with negligible toxicity to breast and colon cancer cells: methyl 4-hydroxy-3,5-dimethoxybenzoate and benzyl 4-hydroxy-3,5-dimethoxybenzoate. The compounds block the proteasome catalytic activity (chymotrypsin-related, trypsin-related, and PGPH), preventing cellular proliferation and promoting programmed cell death [42].

#### 2.1.3. Curcumin 

Curcumin, a chemical found in turmeric, is now a hot topic in cancer research. It has long been utilized in Asian food and medicine, and it is now used worldwide. Curcumin has recently sparked renewed attention, as its numerous therapeutic properties have been discovered in scientific investigations, and it is still extensively employed in India for medical purposes [43]. 

Curcumin is a well-recognized effective natural monomer found in the Zingiberaceae family of plants. It can maintain intracellular Nrf2 by blocking ubiquitin and proteasome. Under standard circumstances, Nrf2 is extracted from the cytoplasm by associating with Keap1, a bridging protein that promotes Nrf2 ubiquitination and consequent protein denaturation by acting as a bridging protein among Nrf2 and the Cullin3/Rbx1/E3 ligase complex. Under stress, the Cullin3/Rbx1/E3 ligase blocks the Nrf2, and Nrf2 aggregates ubiquitination in the nucleus, which leads to an increase in cellular protective genes’ transcription. The stimulation of Nrf2 produced by curcumin is reduced in cells treated with a cysteine 151 mutant Keap1 protein substituted by serine, revealing that serine is the primary target for curcumin-modified Keap1, enabling the secretion of Nrf2. As a result, the curcumin’s unsaturated carbonyl component may be critical for attaching to Keap1 and stabilizing Nrf2 by preventing ubiquitination and proteasomal destruction [44]. 

In glioblastoma (GBM), curcumin modulates the programmed cell death of glioblastoma cells through modifying the protein expression of connexin 43 (Cx43), and thus it can be utilized as an adjunctive treatment. Cx43 protein expression is suppressed by curcumin, although its gene remains unaffected. The cell death produced by TMZ is also exacerbated by curcumin therapy. Curcumin-mediated degradation of Cx43 is inhibited by MG132, a proteasome antagonist, showing that the destruction happens via the ubiquitin-proteasomal cascade. Curcumin is also an autophagy-inducing substance. Autophagy is a type of non-specific breakdown. Ubiquitin is involved in the specific autophagy of ubiquitinated proteins via autophagosomes [45]. 

Curcumin increases BK protein expression in A7r5 cells but does not impact its gene expression, suggesting that BK gene transcription is unaffected but maintains BK protein by lowering its deterioration. Curcumin also boosts BK channel expression and its half-life in HEK293 cells. The proteasome antagonist MG-132 can remove curcumin in both circumstances. Curcumin also promotes BK protein production by suppressing proteasome degradation and triggering the ERK signaling pathway, enhancing BK channels’ function [46].

Curcumin stimulates autophagy and blocks TGF and Smad signal transmission. Suppression of tetrapeptide repeat domain (TTC3) and mediation of ubiquitin of Smad specific E3 ubiquitin regulatory factor 2 (SMURF2) and proteasomal destruction may be involved in Smad 2 and Smad 3 polyubiquitin degradation, limiting the epithelial–mesenchymal transition of hepatocytes [47]. 

EBNA1 modulates Epstein–Barr virus’s (EBV) DNA replication, promoting cellular growth, survival, and cancer, and is a promising approach for EBV-associated ailments. Curcumin decreases the level of EBV nuclear antigen, and hence limits the growth of EBV-related human nasopharyngeal cancerous cells via the ubiquitin proteasomal mechanism [48]. Through NOX4 facilitating the formation of reactive oxygen species, imipramine, in conjunction with curcumin, stimulates proteasomal action and reduces the level of post-translational c-FLIP and MCL-1 in a proteasomal way, inducing programmed cell death by modulating PSMA5 levels [49].

In human cystic fibrosis (CF) bronchial epithelial cells, curcumin lowers the nuclear expression of transcriptional stimulatory protein 1 (SP1), which is among the critical reasons for the enhanced production of basic TLR2. A curcumin-induced SP1 decrease can be reduced using MG-132, a proteasome antagonist. Curcumin is thought to suppress TLR2’s gene abundance and function in CF bronchial epithelial cells by speeding up SP1 destruction through the ubiquitin-proteasome cascade [50]. Curcumin reduces the production of ALKB homolog 5 (ALKHB5), which can enhance the expression of mA-modified TNF receptor-associated factor 4 (TRAF4) mRNA, and so decreases HFD-mediated obesity. As an E3 ubiquitin ligase, TRAF4 increases the denaturation of the differentiating modulator. The PPAR of adipose cells suppress adipogenesis via the ubiquitin-proteasome cascade [51]. 

Curcumin, in conjugation with TNF-associated apoptotic cell death-mediating ligand, can target carcinogenesis synergistically, while causing no harm to conventional proximal renal tubular epithelial cells. The conjugated treatment causes caspase-based cell death, suppresses the proteasome, stimulates the JNK-CHOP cascade driven by ROS, and enables cancerous kidney cells susceptible to TNF-associated apoptotic cell death [52]. Curcumin causes ubiquitination of malignant SIRT1 and consequent proteasomal disintegration to suppress the expression of malignant SIRT1 protein, decreasing the malignancy of human colon cancer cells by irreversibly altering the cysteine 67 motif of SIRT1 [53]. 

It protects PC12 cells by stimulating the proteasome mechanism, whereas rotenone therapy causes significant protein degradation. Curcumin, in conjunction with carfilzomib, a pharmacological proteasome antagonist, triggers apoptotic cell death sensitized to multiple proteasomes, while being harmless to non-cancerous cells. Curcumin is even a potent inducer of the 26S proteasome, which primarily targets DYRK2 and the 26S proteasome, and a specific blocker of bispecific tyrosine regulated kinase 2 (DYRK2). Curcumin inhibits DYRK2-induced phosphorylation of the 26S proteasome. It can enhance the level of p53 and trigger cell death by activating mitochondrial caspases as proteasome inhibitors [54]. RSV (respiratory syncytial virus) is a myxoviridae virus with a negative-stranded RNA that causes bronchitis, asthma, and chronic lower respiratory tract ailments in newborns and young children. It is a potent proteasomal suppressant that reduces RSV replication in Vero cells by inhibiting the stimulation of nuclear factor-kappa B and suppresses degradation of IKB induced by the proteosome [55]. 

In a biphasic way, curcumin and its polyphenolic analog (didemethylcurcumin, CUIII) can influence proteasome functioning. At nanomolar doses, curcumin and CUIII promote proteasomal action, whereas at micromolar values, they decrease it. Curcumin has consistently outperformed CUIII in terms of activity [56]. Curcumin increases the expression of miR-142–3p, while decreasing PSMB5 protein levels, thereby reducing the chymotrypsin-related functionality of the 20S proteasome nucleus. Furthermore, p300, a histone acetyltransferase, can inhibit the level of miR-142-3p [57].

Curcumin was found to effectively reduce the indices of protein ubiquitination in human skeletal muscle in a clinical experiment [58]. Curcumin causes proteasomal dysfunction by binding primarily to the 20S component and blocking chymotrypsin-related, trypsin-related, and peptide glutamyl-peptide hydrolase protease activity. The Thr 1 in the b5 subunit of the proteasome may target the carbonyl carbons in curcumin [59]. Curcumin has some cytotoxicity and adverse effects, despite its favorable pharmacological activities on cells. Both nano-curcumin (5–100 M) and curcumin (10 M), for example, increase the action of proteasomal subunits β2 and β5/β1i, while decreasing 5/1i activity, resulting in enhanced proteasome-induced proteolysis [60]. It effectively suppressed not only chymotrypsin-related action, but also trypsin-related and PGPH-related action in a purified 20S proteasome. Curcumin was discovered to block CT-related functioning in two human colon cancer lines, HCT-116 and SW480, gradually, and to cause programmed cell death in the cells, as indicated by the aggregation of apoptotic proteins. Curcumin was also investigated to determine if it induces G1 arrest, but no significant evidence was found, indicating that proteasomal suppression causes the cells to move into apoptosis. Finally, mice treated with HCT-116 cells had smaller tumors with reduced chymotrypsin-like activity and apoptotic characteristics. Curcumin was given intragastrically every day for three weeks at a dose of 500 mg/kg, resulting in a 40 percent decrease in tumor growth. Curcumin also demonstrated a drop in the indices of cellular proliferation in tumor cells, indicating that it could limit tumor cell growth [59]. Curcumin, like EGCG, has certain challenges with absorption in humans and dissolution rate. Wan, Yang, et al., in 2010, developed curcumin analogues and hydrophilic amino acid conjugates in an effort to address these issues [61].

Curcumin monoacetate and diacetate were synthesized first, and their action on the proliferation of HCT-11 colon cancer cell lines was examined, along with that of curcumin. All compounds demonstrated the same effect on HCT-116 cells. However, curcumin monoacetate showed less efficiency in SW480 cells than the others. The acetates were substantially less powerful in the context of proteasome suppression when CT-like activity was evaluated. The ability of the various curcumin amino acid conjugates to constrain pure 20S proteasome was then evaluated, in addition to the curcumin. The amino acid conjugates were more effective suppressants of the 20S proteasome than unmodified curcumin. The conjugates were then tested in LNCaP prostate cancer cell lines to determine how they affected cellular proliferation. Overall, the findings imply that hydrophilic curcumin amino acid analogs could be a useful strategy to connect curcumin’s chemotherapeutic properties for cancer therapy [61,62].

#### 2.1.4. Celastrol 

Anti-cancer properties of celastrol, a component isolated from a Chinese plant called “Thunder of God Vine,” have been explored in a wide range of investigations. In one such investigation, celastrol was found to be a proteasome inhibitor [63,64,65,66,67]. Celastrol has been used for thousands of years to treat a number of inflammatory diseases, and this study found that it structurally resembles quercetin, a flavonoid that is included in many dietary products. Using computer docking studies, it was put to the test against oridonin, a chemical from a different Chinese plant that was thought to have very little proteasome-inhibiting power. Celastrol stopped the 20S proteasome’s CT-like activity at high concentrations, but oridonin did not have any effect, no matter how much it was concentrated. PC-3 prostate cancer cells that were still alive when celastrol was given, but not when oridonin was given, had a rise in proteins that were targeted by the proteasome. 

Celastrol caused apoptosis, which was shown by a rise in caspase-3 levels and the cleavage of PARP. Celastrol was next examined in LNCaP prostate cancer cells that were positive for androgen receptor (AR) and shown to inhibit the proteasome and reduce the levels of AR [66,67]. This discovery was crucial because of AR’s significance in developing prostate cancer cells. Celastrol was also effective at reducing tumor growth in animals injected with PC-3 prostate cancer cells. Celastrol blocked 65–82 percent of the growth of breast cancer cells. There was a study that found that celastrol and pristimerin could help temozolomide work well on different types of melanomas cell lines [66,67,68,69]. Both combinations of treatments worked better than temozolomide alone at controlling cell growth in the SK-MEL-173 cell line. Celastrol and temozolomide worked better together, when they were used together in four other resistant melanoma cell lines. Each line had a lower IC_50_ for temozolomide. They also looked into whether celastrol could stop the proteasome in SK-MEL-173. A chemical called celastrol was put into the cells. Then, Western blot analysis was performed on the cells to look for ubiquitinated proteins. However, there were more IKB and ubiquitinated proteins, which meant that the proteasome was slowed right down because it took longer to break down. In the first step, IKB was phosphorylated, which means that NFKB can be activated. This makes genes in the nucleus more likely to be turned on and off. This study also looked into how NFKB and celastrol work together in the same way. Celastrol and temozolomide were both used to treat melanoma cells. When celastrol was used first, followed by temozolomide, the phosphorylation of IKB was blocked, which further blocked the activation of NFKB. There were no changes in the level of NFKB expression. A crucial stage in NFKB’s ability to influence gene expression was its inability to travel to the nucleus. If this is true, it could help us understand celastrol’s role in slowing cell growth in melanoma lines previously resistant to the drug [67,69].

#### 2.1.5. Pristimerin 

Another organic compound derived from Chinese plant *Celastrus hypoleucus* has been investigated for its ability to treat cancer. Pristimerin is the methyl ester of celastrol, and it has also been used in cancer treatment research. In docking investigations, pristimerin, which is also used to treat inflammation, was extremely potent. Prostate cancer cells such as PC-3, and numerous other cell lines with high AR levels, experience reduced AR levels in response to celastrol. Inhibition of the proteasome by pristimerin caused cell death via interfering with the AR signaling pathway [67,70].

#### 2.1.6. Triptolide

Another component obtained from the Chinese “Thunder of God Vine” is triptolide, inhibiting the proteasome. Triptolide inhibited cell proliferation successfully in both MDA-MB-231 breast cancer and PC-3 prostate cancer cells. Breast cancer cells were marginally more amenable to therapy than PC-3 cells [67,71]. Triptolide was able to efficiently inhibit the proteasome’s CT-like activity while the PC-3 cells were still alive. This occurred in a dose- and time-dependent manner. Additionally, it destroyed both MDA-MB-231 and PC-3 cells but was more effective against breast cancer cells. Triptolide does not appear to inhibit the function of the purified proteasome, but it can inhibit the proteasome in living cells. To begin, the triptolide molecule may be unable to attach to the 20S proteasome’s β5 subunit due to certain linkages in it. Second, triptolide may inhibit the activity of other components of the 26S proteasome, such as the 20S subunit. The researchers propose that if triptolide is adjusted appropriately, it may be extremely effective at combating cancer [67,71].

#### 2.1.7. Shikonin

Shikonin is a naphthoquinone chemical obtained from the root of “*Lithospermum erythrorhizon*”, a plant that has been used in Chinese medicine for centuries to heal disease-related cuts, sores, and burns. Today, it has been demonstrated that when cancer cell lines are treated with it, they undergo apoptosis. There is currently no understanding of how shikonin induces apoptosis. At various dosages, the researchers discovered that shikonin inhibited the 26S proteasome in both human PC3 prostate cancer cells and murine hepatocellular carcinoma H22 lines [67,72]. Shikonin also induced apoptosis in these cell lines. Additionally, it has been proven that the proteasome is inhibited before cell death. On mice, an in vivo experiment was conducted using P388 leukemia cells. Only one shikonin-treated animal survived the 28-day experiment, but all control mice died after 23 days [67,69,72].

#### 2.1.8. Withaferin A

*Withania somnifera*, commonly referred to as Indian winter cherry, produces withaferin A, a steroidal lactone. For centuries, traditional medicine practitioners in Southeast Asia have used the plant to cure everything, from tumors and wounds to age-related illnesses. Additionally, withaferin A has been researched for its anti-cancer properties to understand its efficacy and mode of action better. According to a study by Yang, Shi, et al., in 2007, withaferin A suppressed the 20S proteasome effectively, with the β5 subunit as its principal target [67,73,74]. In computer docking experiments, withaferin A was discovered to attach to the 20S proteasome’s β5 subunit, inhibiting its CT-like activity. When examined in vitro, purified 20S proteasome was found to inhibit CT-like activity. When withaferin A was used to treat PC3 prostate cancer xenografts, the results were identical. In these cells, apoptosis was also detected, as demonstrated by Western blotting for indicators of apoptosis. Proteasome inhibition by withaferin A increased PARP and caspase-3, as along with morphological alterations in the cells.

Similarly, in LNCaP cells, ubiquitinated proteins were elevated, and androgen-receptor expression decreased over time. Finally, a mouse xenograft of a human PC-3 was generated. Withaferin A-treated mice had tumors that were significantly smaller than those of the control mice, and one of the treated mice was completely tumor-free. Proteasome-targeted proteins such as IκB-α, p27, and Bax were found to have enhanced ubiquitination in the tumor tissues examined after the experiment [67,74].

#### 2.1.9. Gambogic Acid

It is the principal pigment of gamboge resin from various *Garcinia* species. It has been used for centuries in traditional Chinese medicine to cure human diseases, including cancer. Chinese regulators have approved gambogic acid (GA) in clinical trials to treat a range of cancers [75,76]. Numerous putative targets for GA have been revealed, but its molecular targets remain a mystery. GA is equally as effective as bortezomib at inhibiting tumor proteasome activity, but with a lower risk of side-effects. Additionally, intracellular CYP2E1 metabolizes GA to provide it with proteasome-inhibitory properties. There is a lot more CYP2E1 gene expression in tumor tissues than in many normal tissues, which leads to tissue-specific proteasome inhibition and toxicity for cancers. Various cancer cell types have shown these effects to be effective [77,78].

#### 2.1.10. Resveratrol 

It is a well-known phytoestrogen found in red wine and among other foods. Despite its various health-enhancing characteristics, its effect on HER2+/Erα breast cancer cells appear to be significantly unfavorable. The resveratrol-induced accumulation of ∆16HER2, the production of ∆16HER2/HER heterodimers, and the stimulation of the mTORC1/p70S6K/4EBP1 cascade all contribute to the rise and proliferation of cancer cells, in both in vivo and in vitro settings [79,80,81,82]. Many studies have shown that proteasome-inhibiting can have a beneficial or negative effect, depending on the disease entity (particularly the type of malignancy). There is evidence that resveratrol can reduce the activity of NF-kB and the TNF-α protein, which are both important players in inflammation in neurodegenerative and cardiovascular illnesses, and in many other inflammatory conditions [82,83,84]. Glioma stem cells (GSCs; the chemotherapy-resistant cells responsible for tumor growth and relapse) are less able to self-renew and initiate (form, multiply) tumors in glioblastoma cells when resveratrol is present. Activation of the p53/p21 pathway and reduction of NANOG through proteasomal degradation are both caused by resveratrol, and this suggests a treatment method for glioma that could be used [82,85].

#### 2.1.11. Quercetin

The flavonoid quercetin can be found in onions, apples, citrus fruits, and green leafy plants. It has potent antitumor activity, which is multidirectional. Additionally, there is evidence that it possesses anti-inflammatory effects. Quercetin suppresses the activity of all three catalytic subunits of trypsin-like (T-L), proteasomal chymotrypsin-like (ChT-L), and caspase-like/peptidyl-glutamyl peptide-hydrolyzing-like (PGPH) enzymes, and the ChT-L subunit has the largest inhibitory impact [82,86,87]. Quercetin causes apoptosis in cancer epithelial cells because it stops the activity of the proteasome and makes more polyubiquitinated proteins. This flavonoid also causes autophagy by stopping mTOR from working. Quercetin is also more effective than kaempferol or myricetin at blocking the 26S and 20S proteasomes in Jurkat T cells. This is because quercetin is an antioxidant [82,88,89]. It was found that a drug’s capacity to inhibit the proteasome depended on the number of OH groups it contained. The more OH groups there are, the more effective it is. Additionally, the quantity of hydroxyl groups in flavonoids has a significant effect on how antioxidants and cells function. The presence of an unsaturated bond in the C-ring of flavonoids can also affect their ability to prevent toxins from entering the body. This is demonstrated by apigenin, which has an IC_50_ value 21 times that of naringenin, which lacks an unsaturated bond in the center. Unsaturation on carbon C4 makes the compound more active. The threonine β5 subunit’s N-terminal end can be attacked by nucleophiles, making it more active. In contrast, the presence of saturated ring C makes it less effective at blocking [82,89,90]. It may also be because quercetin and kaempferol have aromatic ketone groups that are easy for nucleophiles to attack. This could be why the proteasome’s ChT-L activity is slowed down [66,82].

#### 2.1.12. Genistein

Genistein, an isoflavone present in soybeans, has been shown to stop the development of human cancer cells and greatly reduce the chance of developing hormone-dependent cancers. It has been demonstrated to cause apoptosis that is p53-dependent in previous studies [82,91,92,93]. The anti-chymotrypsin-like activity of proteasome has been demonstrated to be inhibited by this polyphenol both in vitro and in vivo. The in vitro activity of genistein is mediated via interaction with the proteasome subunit β5. IkB, Kip1, and the pro-apoptotic Bax protein become ubiquitinated as a result, and apoptosis is subsequently triggered. Healthy cells do not undergo proteasome inhibition or apoptosis; only malignant fibroblasts do. The mechanism through which genistein affects tumor cells is mainly by targeting the topoisomerases II (Top II) [82,94,95,96]. A drug called doxorubicin, for example, is used to fight cancer because it damages DNA by attacking Top II. According to a study, the proapoptotic effect of genistein on HeLa cells may be due to a decreased transcription level. However, there is considerable evidence that Top II inhibitors have a significant likelihood of producing translocations that result in secondary malignancies [97].

#### 2.1.13. Kaempferol

Kaempferol, a flavonol present in various foods, viz., tomatoes, grapes, apples, green tea, and broccoli, is a popular compound. It has been shown to have a variety of qualities, including prooxidative and antioxidant effects, based on the quantity in the cell and the environment. It exhibited several properties, including prooxidative activity (greater production of ROS in glioblastoma cells), anticancer activity (suppression of, among others, RSK2, Src, p53 action, suppression of Akt activity, and suppression of ERK activity), and antiangiogenic action [82,98,99,100]. Kaempferol has been demonstrated to inhibit the degradation of MAGEA6, a ubiquitin ligand for AMPKα1. It is believed that a deficiency of AMPKα1 in the body could result in the development and destruction of human DNA.

Additionally, it makes human glioblastoma cells more susceptible to TRAIL-induced apoptosis, as survivin is degraded by the proteasome. When Akt is inactive, survivin becomes less stable. This occurs because the phosphorylated kinase stabilizes survivin, making it more lasting [82,101,102,103]. Another possible reason the genome does not stay stable is when there are changes to DNA methylation. DNMT methyltransferases are important to the process of DNA methylation. Qiu and his colleagues discovered that kaempferol promotes the proteasomal pathway in bladder cancer cells, activating the DNMT3B methyltransferase degradation process. PI3K and Akt signals regulate the proteasomal degradation of DNMT3B; and p-Akt inhibits the degradation of DNMT3B when it interacts with it. Several previous investigations have demonstrated that kaempferol inhibits the PI3K/Akt pathway, increasing the ubiquitination of DNMT3B and making it simpler to eliminate [82,104].

#### 2.1.14. Green Tea Polyphenols 

**Figure 3:**
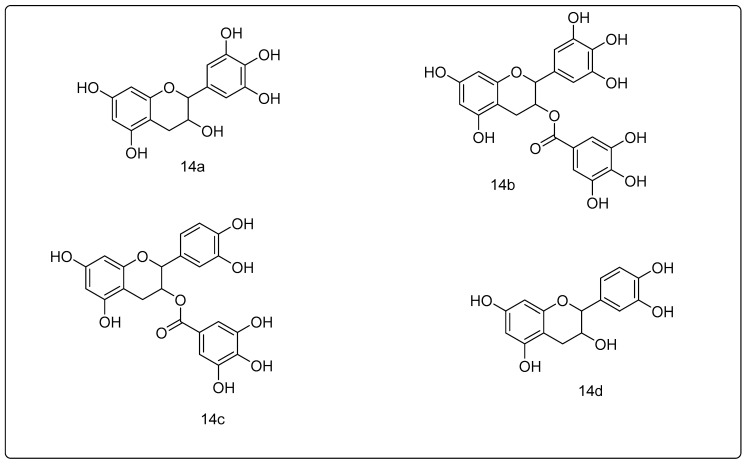
Green tea polyphenols: (**14a**) epigallocatechin; (**14b**) epigallocatechin gallate; (**14c**) epicatechin gallate; (**14d**) epicatechin.

The main polyphenols in the tea plant are epigallocatechin (EGC), epigallocatechin-3-gallate (EGCG), epicatechin 3-gallate (ECG), and epicatechin (EC) (Figure 3), and their epimers. Both the 5 and 1 catalytic subunits of the proteasome were shown to be inhibited by EGCG. The identical chemical, on the other hand, is unable to suppress the two subunits [105,106,107,108,109,110,111,112,113]. At 80–200 nM, EGCG inhibits proteasomal activity in cancer cells in vitro. The effective doses are necessary for proteasome suppression to rise to the 1–10 μM range in vivo, which corresponds to the range of concentrations reported in serum, following green tea ingestion [110]. EGCG has high selectivity for cancer cells, as it has a minor inhibitory effect on non-cancer cells’ proteolysis [114]. The capacity of laboratory synthesized EGCG amides and analogs utilizing changed ester linkages and rings to suppress the five subunits of the proteasome is similar to that of the natural substance [113]. The ester bonds present in EGCG, EGC, ECG, and EC appear to be decisive of their proteasomal antagonistic capacities, as the latter are not correlated with GCG (gallocatechin 3-gallate), GC (gallocatechin), CG (catechin-3-gallate), and C (catechin), or their relating epimers, which lack the aforementioned ester bonds. Lactacystin, the most extensively used proteasome antagonist, has an ester link in its active state (lactone) [110]. 

Manufactured compounds of tea polyphenols have previously been patented for proteasome suppression and cancer therapy [110]. EGCG is unstable under physiological circumstances, irrespective of its effectiveness as a proteasome antagonist. It is suggested that in vivo biotransformation mechanisms such as methylation diminish its capacity to suppress the proteasome. As a result, attempts have been undertaken to improve its bio-absorption by developing prodrugs that demonstrated promising outcomes in vitro in cell-free and cellular mechanisms, exhibiting greater effects than the known natural molecule [109,115]. With the aid of a proteasome denaturation cascade, EGCG decreases the accumulation of pulmonary fibrosis-associated mutant surfactant protein-A2 (SP-A2), but the proteasomal antagonist MG-132 can counteract the EGCG-mediated aggregate diminution [116]. 

Cannabinoids cause the Rap1GAPII proteasome to be degraded by G (alpha) o/i, lowering its integrity and activating Rap1, resulting in neurite development. Proteasome antagonists can prevent Rap1 activation caused by this mechanism [117]. Gallic acid reduces the amount of EGFR via speeding up EGFR transformation, causing death in EGFR mutant non-small cell lung cancer cells (MSCLC), although proteasome antagonists can counteract this action [118].

Green tea is rich in polyphenols, which have anti-inflammatory and anti-cancer effects. Epicatechin (EC), epigallocatechin (EGC), epigallocatechin-3-gallate (ECG), and epigallocatechin-3-gallate (EGG) are all anticancer chemicals found in this famous drink. EGCG, which has the greatest antineoplastic effects among the green tea components, is a component of particular interest [119]. EGCG is anti-proliferative (it is believed that it is potent than 5-fluorouracil, which is employed to manage colon cancer), pro-apoptotic, anti-angiogenic [120], and anti-neoplastic [119].

The efficacy of EGCG in the management of malignancies of the gastrointestinal tract, ovarian, breast, prostate, pancreatic, and lung cancer has been proven by epidemiological studies [121]. Green tea extract inhibits glycosylation in the endoplasmic reticulum, altering protein maturation post-translationally in vivo and in vitro [122]. EGCG kills Jurkat T leukemic cells. However, this compound’s great effectiveness is obtained by delivering it as a six-fold better prodrug (peracetate (-)EGCG) which hydrolyzes and releases EGCG once it reaches the interior of the cell [123]. EGCG, on the other hand, is an efficient proteasome inhibitor when taken as a pure molecule at quantities accessible in human blood [124]. 

Polyphenols with an ester linkage, such as ECG, GCG, and CG, were shown to inhibit the proteasome’s chymotrypsin-like activity in vivo and in vitro. EGCG (in vitro: IC50: 86–194 nm, in vivo 1–10 mM) has the strongest inhibitory action of these compounds, which has not been established by substances that do not include ester bonds: EC, GC, and C [124]. Purified 20S proteasomes in tumor cell extracts and 26S in intact cells are all strongly inhibited by EGCG. Moreover, the function of phenylalanine coupling to the hydrophobic S1 pocket of the proteasome’s b5 subunit is mimicked by EGCG’s A ring [110,125].

EGCG is a gallic acid and epigallocatechin ester with a unique structure. There are four rings in total: A, B, C, and D. A benzopyran moiety with a phenyl group in the C2 position and a gallic acid group in the C3 position are represented by rings A and C. The ester link renders the molecule vulnerable to nucleophilic action, and the hydroxyl group of the D ring allows hydrogen bonds to form with the proteosome’s Gly47 and Ser131, perhaps stabilizing the interaction between the proteasome and ()-EGCG [126].

The buildup of proteasome substrates such as p27Kip1 and IkB-a, and the suppression of the cell cycle in the G1 phase, occur when the proteasome is inhibited by EGCG [124]. Recent research suggests that EGCG has a significant anti-tumor efficacy through modulating b-catenin expression levels in the management of head and neck cancer. In in vivo malignancies, modifications in b-catenin transcription, translation, and degradation have been identified. EGCG promotes b-catenin ubiquitination and proteasomal destruction while also lowering the expression levels of Akt, GSK-3b, and cyclin D-1 proteins [127].

EGCG has been shown to suppress the levels of 19S and 20S proteasomal proteins and drastically diminish the chymotrypsin-like activity of proteasomes in experimental rats [128]. As ubiquitin ligases show a vital function in signal transduction and some are highly expressed in cancerous cells, inhibiting them in anticancer treatment is also significant. EGCG can actively interact with the E3-TRAF6 ubiquitin ligases Gln54, Gly55, Asp57 ILe72, Cys73, and Lys96; diminish the interconnection between TRAF6 and UBC13 (E2); hinder the signaling pathway of p65 and p50; and inactivate the NF-kB signaling pathway, thereby inhibiting melanoma’s cell growth, migration, and invasion [129]. This polyphenol boosts the gene transcription of p53, among the most significant tumor suppressors, by phosphorylating it at Ser15 and Ser20. However, in normal settings, as p53’s concentration rises, so does the expression level of mouse double minute 2 homolog (MDM2) (E3 ligase), which is accountable for polyubiquitinating p53, making it intended for destruction, as a consequence, reducing its concentration. Jin et al. discovered that EGCG affects p53 aggregation and enhances the protein’s stability via compromising the association between p53 and MDM2 [130].

### 2.2. Marine-Derived Proteasome Inhibitors

#### 2.2.1. Carmaphycins

**Figure 4:**
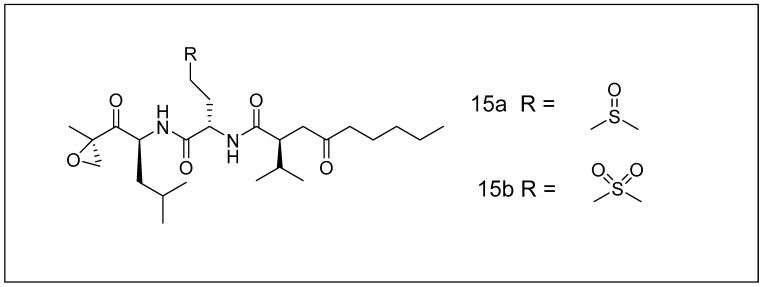
Chemical structures of carmaphycins **15a** and **15b**.

Isolation of carmaphycins was recently performed using the species “*Symploca*,” cyanobacteria from the sea [131]. They are identical to epoxomicin and its derivatives, a series of proteasome antagonists exhibiting cytotoxicity and cytostatic properties because they are β-epoxyketones [131]. Figure 4 depicts the chemical structure of carmaphycins A and B. Carmaphycin blocks the action of 20S proteasome with an IC_50_ of about 1.5 nM (chymotrypsin-like) and with an IC_50_ of 46 nM (trypsin-like). Interestingly, with IC_50_ values less than 20 nM, it has been reported to restrict the proteasome from working in the lungs and colon cancer cells (H-460 and HCT-116, respectively) [132].

Natural carmaphycins and their synthetic counterparts were developed for the proteolytic suppression of any chymotrypsin-like protease, whether it is used to treat cancer, neurological disorders, or retroviral infection [133].

#### 2.2.2. Aaptamine and Its Derivatives

**Figure 5:**
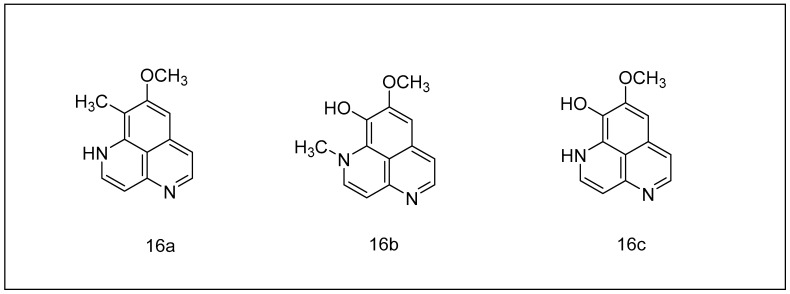
Aaptamine and its derivatives: (**16a**) aaptamine, (**16b**) isoaaptamine, (**16c**) demethylaaptamine.

Aaptamine, an alkaloid, was first discovered in the marine sponge Aaptos in 1982 [134]. Figure 5 depicts aaptamine, and its natural variants, isoaaptamine and demethylaaptamine. These alkaloids are most predominantly reported in species of the Hadromerida order, though they have also been detected in a Haplosclerida sponge [135]. The efficacy of several molecules at suppressing both chymotrypsins and caspase-type regulatory sites on the 20S proteasome has already been shown to have an antitumor effect. Demethylaaptamine has an IC_50_ of 10 M overall chymotrypsin-like protein inhibition and around 9.5 M caspase-like inhibition in 20S proteasome. The IC_50_ for isoaaptamine reached 7.45 M for both sites, whereas the IC_50_ for aaptamine reached 18.84 M for chymotrypsin and 20.15 M for the caspase [136,137]. 

#### 2.2.3. Salinosporamides

**Figure 6:**
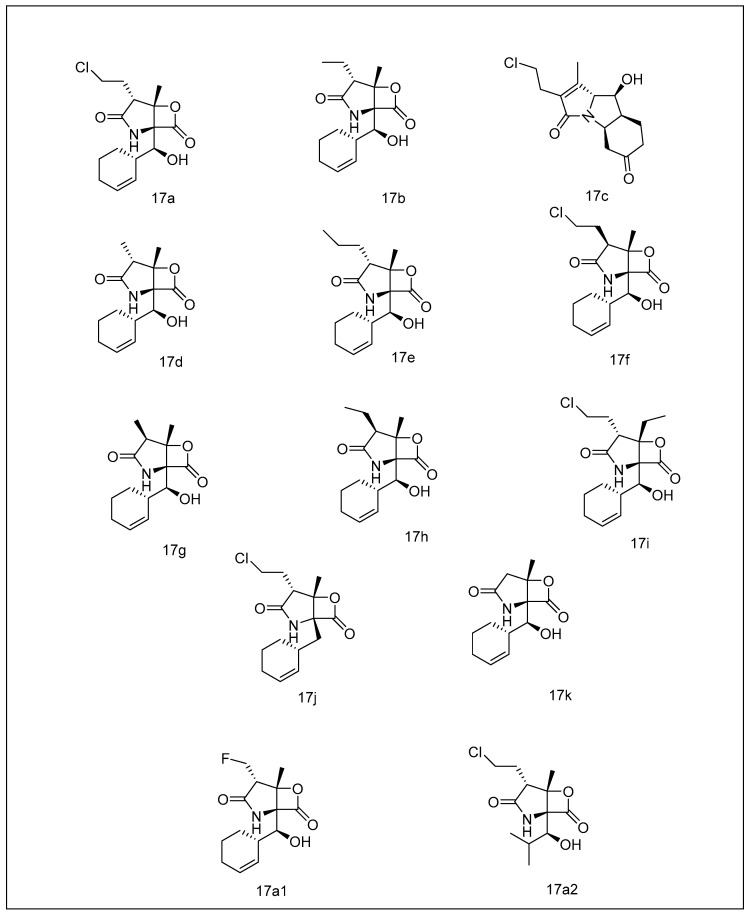
Salinosporamides with their structural counterparts: (**17a**) salinosporamide A; (**17b**) salinosporamide B; (**17c**) salinosporamide C; (**17d**) salinosporamide D; (**17e**) salinosporamide E; (**17f**) salinosporamide F; (**17g**) salinosporamide G; (**17h**) salinosporamide H; (**17i**) salinosporamide I; (**17j**) salinosporamide J; (**17k**) salinosporamide K; (**17a1**) fluorosalinosporamide; (**17a2**) antiprotealide.

Salinosporamides, with their structural counterparts (salinosporamides B–K) (Figure 6), are a class of natural compounds produced by Gram-positive coastal actinomycetes belonging to the genus Salinospora. Salinosporamide A (**17a**) was discovered in *Salinospora tropica* for the first time [138,139]. It blocks every one of the key catalytic sites of the 20S proteasome, with an IC_50_ of 1.3 nM against the chymotryptic subunit. The trypsin-like and caspase-like active sites have IC_50_ values of 2.6 and 430 nM, respectively, for the other sites [138,139]. It also has antitumor action at nanomolar concentrations [139]. Salinosporamides and a salinosporamide A analog have been patented as anticancer drugs [140,141]. An additional patent for salinosporamide analogs with modified heterocycles has also been filed [133]. However, its leading pro-apoptotic impact is mediated via an extrinsic pathway which involves increased Smac release and transactivation of caspase-8 and caspase-9 [142].

Salinosporamide A is the most efficient salinosporamide and has the ability to block the proteasome at low nanomolar concentrations. Salinosporamide C (**17c**) (decarboxylated pyrrole alternative), salinosporamide I, (**17i**) (a C-3 ethyl alternative), and salinosporamide J (**17j**) (non-hydroxylated analog) are the only natural variations of (**17a**) that differ primarily in C-2 side-chain modifications. The bioactivity of natural and synthetic salinosporamide analogs was assessed to comprehend their modes of activity and highlight structure–activity relationship (SAR) characteristics completely. As per SAR investigations, at the C-2 side chain, a good releasing group is required for strong action against every β subunit, because it aids the tetrahydrofuran (THF) ring—closing those preserves the proteasome–inhibitor pair [143]. Good releasing groups, notably, halogens (Cl-, Br-, I-) and sulfonate esters (dansylate, mesylate, and tosylate), seem to be well accepted and fit well into the proteasome’s wide S2 binding domain. As an outcome, non-leaving groups substantially lower therapeutic effectiveness. Such results were verified by the intriguing example of fluorosalinosporamide (**17a1**), created in B. S. Moore’s research laboratory by genetic alteration of the parent cell to add fluoride rather than chloride as that of the releasing group [144]. Fluoride’s weak releasing potential and resultant F-atom sluggish migration upon proteasome-inhibitor contact is consistent with the action of fluorosalinosporamide like a partly reversible inhibitor, validating the above mode of action.

Thus, fine-tuning proteasome blockage by salinosporamides is possible by modifying the releasing group’s ability at C-2. Furthermore, the biological compound’s C-2 stereochemistry must be preserved to maintain robust bioactivity, since salinosporamide F, (17f), the original C-2 epimer of (**17a**), is a hundred-fold less active [143,145]. Except for the synthetic variant, cyclopentenyl, which demonstrated bioactivity identical to (**17a**) in terms of cyclohexenyl unit substitutions at C-5, every C-5 homolog produced diminished activity [146]. Antiprotealide (**17a2**) is a tiny metabolite of *Salinospora tropica*, including an isopropyl sequence at C-5 and even fifty-fold lesser inhibition than (**17a**). However, at C-5, a linear side chain lowered the bioactivity, most likely because of the destabilizing “flapping” function of a quite adaptable chain inside this proteasome’s S1 linker [146]. Subsequent modifications to the original scaffold (**17a**), such as salisporamide I (**17i**) at C-3, the replacement of methyl or even salisporamide J (**17j**) at C-5, and the omission of hydroxy, are always related to decreased bioactivities [145,146,147]. 

SAR analyses revealed salinosporamide A, the most potent antagonist of proteasome compared to natural and conventional variants, emphasizing a structure that was ideally built by nature for every atom in the right spot to enhance interactions with the target [148]. **17a** has shown maximized cell death, restricted osteoclast genesis-constrained penetration via NF-κB route destruction, and NF-κB-Snail-RKIP interruption, resulting in suppression of antiapoptotic genetic variants and conversion to mesenchymal from epithelial cells [149,150,151]; and it initiated caspase-8 and ROS-dependent apoptotic cell death in leukemia [152]. Preclinical investigations revealed that **17a** had higher efficiency (significantly greater potency and prolonged duration of bioactivities) than bortezomib [149], and this, coupled with lower toxicity at recommended doses, was designed to allow (**17a**) one to access the clinical development cycle in 2006 with a phase I trial (NCT00396864) in individuals having high-risk tumors and refractory lymphoma, whose illness had evolved after therapy modalities. Subsequently, four more phase I/II trials have been accomplished to investigate the safeness, pharmacokinetic properties, and even pharmacodynamics of **17a** in individuals having (a) regressed or persisted multiple myeloma (NCT00461045); (b) strong tumors, chronic lymphocytic leukemia (CLL), lymphoma, regressed or relapsed (RR) multiple myeloma (NCT00629473), and Waldenstrom’s macroglobulinemia; (c) combining **17a** with HDAC antagonist vorinostat (NCT00667082) in pancreatic carcinoma and melanoma; (d) pomalidomide combining with dexamethasone (low-dose) for regressed and refractory multiple myeloma (NCT02103335) [153,154,155]. 

Salinosporamide A had also been found to have a particular cytotoxic effect on malignant glioma stem cells and glioma cell lines, having little or no impression on neural stem cells [156]. As a result, three clinical trials evaluated (a) different combinations of **17a** and the drug bevacizumab (BEV; Avastin^®^, Genentech Inc., South San Francisco, CA, USA), employed for the therapies of malignant glioma (WHO Grade IV) (NCT02330562) (b) individuals with newly diagnosed malignant glioma (WHO Grade IV), combining 17a with radiation therapy (RT), optune (NCT02903069), and temozolomide (TMZ); (c) the efficiency of (**17a**) in individuals with freshly detected glioblastoma (phase III trial, NCT03345095). As marine-derived compounds are generally extracted in trace concentrations from their native sources, their complete utilization for therapeutic development is usually hampered. To overcome such issues and secure the creation of (**17a**), efforts were directed toward optimizing a commercial saline brewing process that adhered to Current Good Manufacturing Practice (CGMP) norms. Despite the fact that more than ten synthetic ways to create one have been proposed over the years, the substance was just now obtained via cultivating the naturally producer *Salinospora tropica* [157]. This is the first time a GMP saline fermenting technique has been used to make a natural-product medication from such a marine microbe, indicating that new drug utilizing marine natural compounds is a viable option.

### 2.3. Terrestrial-Derived Proteasome Inhibitors

#### 2.3.1. Lactacystin

**Figure 7:**
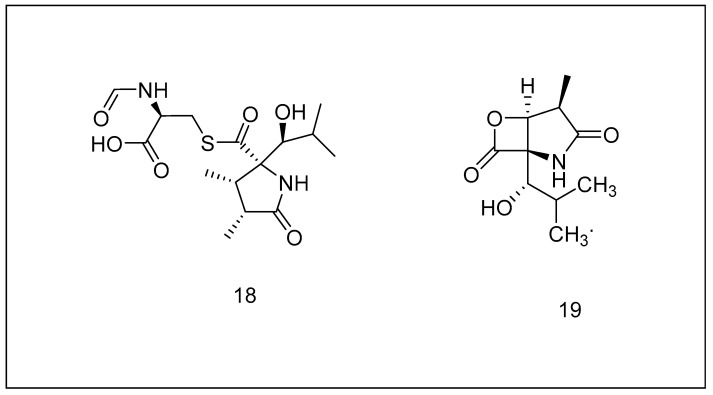
Chemical structures of (**18**) lactacystin and (**19**) clasto-lactacystin b-lactone.

This naturally occurring substance was produced by the streptomyces bacterium. Lactacystin has been used as a research tool in proteasome investigations, when it was discovered that it could reversibly suppress PGPH-like and permanently block active domains such as trypsin and chymotrypsin for 20S proteasome. Lactacystin achieves this by altering the hydroxy (side chain) of the amino-end threonine in at least one of several catalytic subunits of the proteasome [158,159]. In addition, this chemical, coupled to omuralide (clasto-lactacystin-β-lactone), has been shown to block additional proteases, including lysosomal cathepsin A [160]. Figure 7 illustrates the structures of lactacystin (18) and clasto-lactacystin-β-lactone (19). Numerous investigations have been investigated to assess the ability of lactacystin to prevent infectious diseases by viruses, which rely just on host cells’ UPS to complete their tasks, and it has been discovered that this molecule can prevent Japanese encephalitis illness, along with human immunodeficiency virus infection at an initial stage [161]. This compound was utilized to investigate the detrimental effects of proteolysis dysfunction in neurodegeneration. It was discovered that proteasome inhibition could disrupt calcium homeostasis, raising concerns about the probable adverse effects of these compounds [162]. Lactacystin has since been utilized to imitate the conditions of neurodegenerative disorders in neuronal cell cultures [163]. Patents endorse the pharmaceutical uses of these substances, and some of their synthesized derivatives [133], and there are even approaches for manufacturing these variants and clasto-lactacystin-lactone on its own [164].

#### 2.3.2. Ubistatins

**Figure 8:**
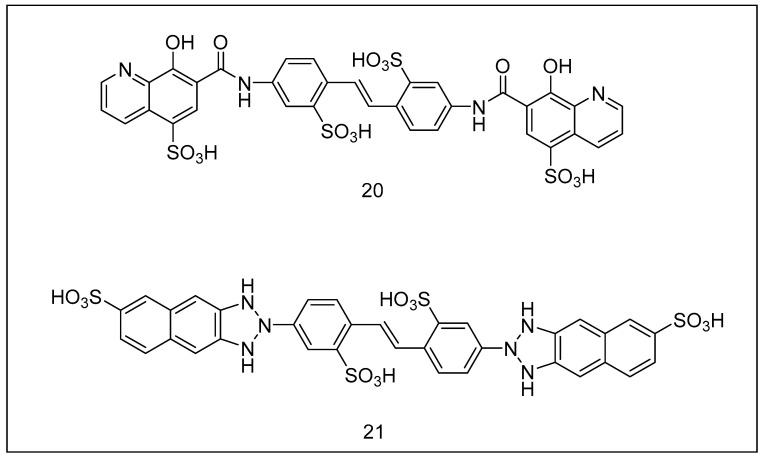
Chemical structures of (**20**) ubistatin A and (**21**) ubistatin B.

Ubistatin (Figure 8) was just the name given to a group of alkaloids discovered lately in extracts of Xenopus specimens [165]. Ubistatin A is the most effective of these chemicals (ubistatin A (20) ubistatin B (21)). It inhibits deubiquitination with an IC_50_ of 400 nM, disrupting the proteolytic process of the proteasome by impairing the attachment of ubiquitinated proteins to the 26S proteasome [165,166]. As a result of these processes, proteolysis is impaired, which contributes to the induction of apoptosis due to cyclin B-degradation suppression. These chemicals may effectively overcome resistance to various proteasome antagonists since they target upstream proteolytic degradation.

#### 2.3.3. Epoxomicin

**Figure 9:**
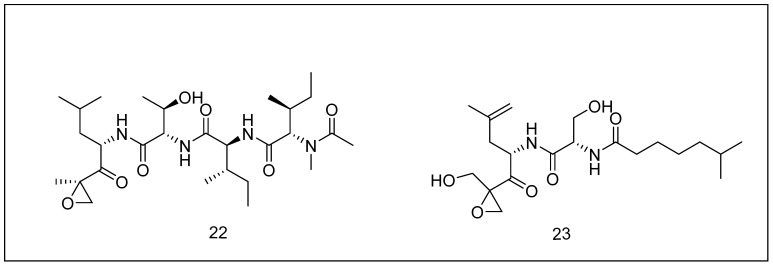
Chemical structures of epoxomicin (**22**) and eponemycin (**23**).

In 1992, epoxomicin was first obtained from strain actinomycete, Q-996-17, and was found to have extreme cytotoxicity to numerous cancer cell lines. The strain was discovered in mud trials in India and is thought to be an actinomycete from the genera Thermomonospora. However, the species is unknown. Epoxomicin belongs to the same eponemycin family as eponemycin [167]. When it was first discovered, eponmycin was found to have a substantial cytotoxic effect on multiple cancer cell lines, but so far, no antifungal or antibacterial action has been observed [168]. Figure 9 illustrates the structures of epoxomicin (**22**) and eponemycin (**23**).

Carfilzomib (Kyprolis^®^), a proteasome antagonist developed from epoxomicin, is now used in the management of regressed/relapsed multiple myeloma and mantle cell lymphoma. Given that, bortezomib, an often used proteasome inhibitor, and carfilzomib, the very first naturally sourced antagonist of the proteasome, has been permitted by the FDA (in 2012) [169,170].

#### 2.3.4. Syrbactins

**Figure 10:**
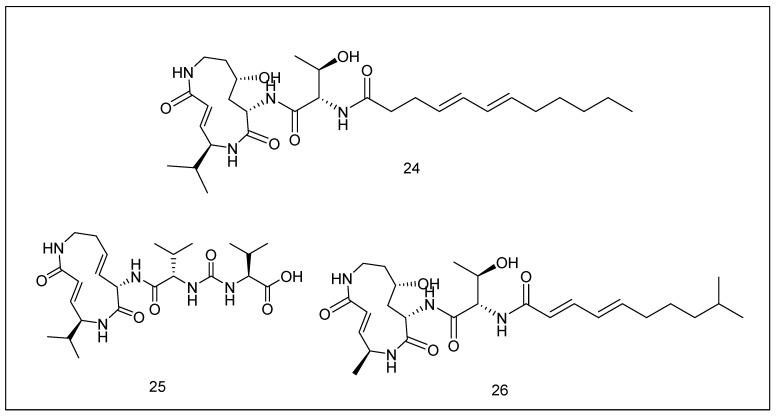
Chemical structures of the glidobactin (**24**), syringolin A (**25**), and cepafungin I (**26**).

Syrbactins that included glidobactin A (**24**), syringolin A (**25**), and cepafungin I (**26**) have comparable chemical structures (possess a 12-ring structure) and biosynthesis processes and all have the ability to block the proteasome [171,172] (Figure 10).

In terms of syrbactins’ modes of action, syringolin A has been found to irretrievably restrict all three key catalytic domains in the eukaryotic 20S proteasome, though suppression of the β2 subunit is minor, because this component has a Ki’ of 843 nM against the five subunits and a Ki’ of 6.7 µM against the β1 subunit. The β5 subunit is the most sensitive [172,173]. The Ki’ differentiates from the IC_50_ in that it provides more information about the concentration required to interact with 50% of the targeted protein, irrespective of inhibiting capacity [174]. Glidobactin A, on the other hand, cannot inhibit the β2 subunit of the proteasome but may irreversibly inhibit the β5 subunit (Ki’ of 49 nM, the greatest antagonist of this site amongst syrbactins), and to a lesser extent, the β2 (Ki’ of 2 µM) [172]. Syrbactins cause apoptosis and autophagy in neuroblastoma cells, resulting in cell death. p53 and Akt/PKB are triggered simultaneously, which fits the profile of an appropriate proteasome antagonist for cancer treatment [171]. Apoptosis-inducing action of these compounds has been found in neuroblastoma cells (SK-N-SH and LAN-1) and in SKOV3 (ovarian cancer) cells [175,176,177]. 

#### 2.3.5. Tyropeptins

**Figure 11:**
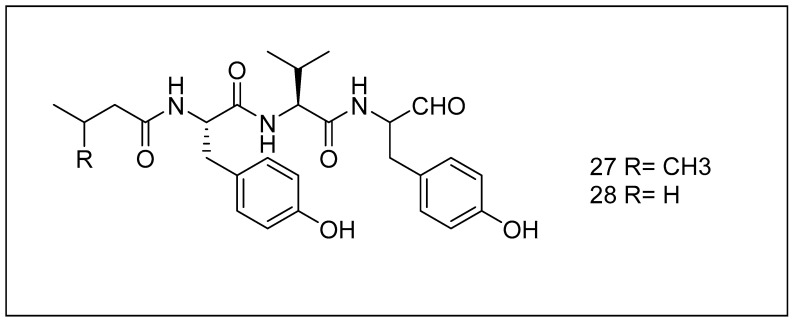
Chemical structures of tyropeptin A (**27**) and tyropeptin B (**28**).

Tyropeptins A (**27**) and B (**28**) (Figure 11) were first extracted from the soil actinobacteria *Kitasatospora* sp. [178,179]. Tyropeptin A inhibits the proteasome most effectively. It has a substantial inhibitory effect against the proteasome’s proteolytic binding domains, such as chymotrypsin (IC_50_ of roughly 20 nM) and trypsin (IC_50_ of 2.93 µM), despite being inept at blocking the action, like caspase of the proteasome in vitro [178]. Tyropeptin A inhibits proteasomal action and is identical to entire cells [180]. The IC_50_ of these compounds for inhibiting the site like chymotrypsin of 20S proteasome is as minimal as 1.4 nM [181].

## 3. Compounds Inhibiting the Proteasome in Clinical Trials

### 3.1. Bortezomib

**Figure 12:**
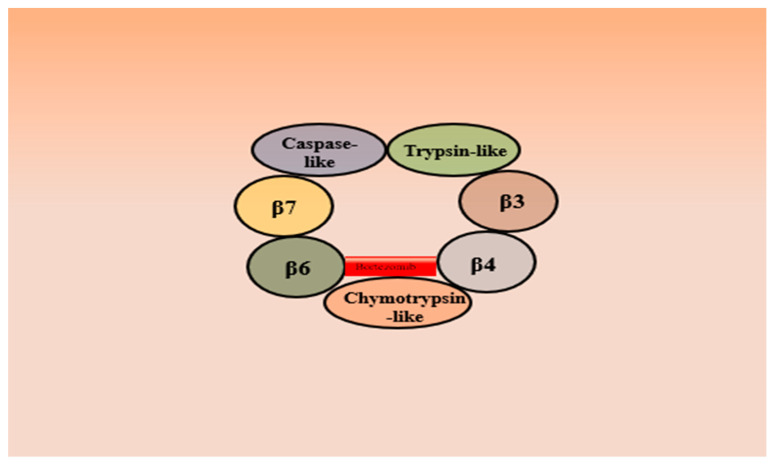
Cross-sectional view of bortezomib’s binding site in the proteasome.

**Figure 13:**
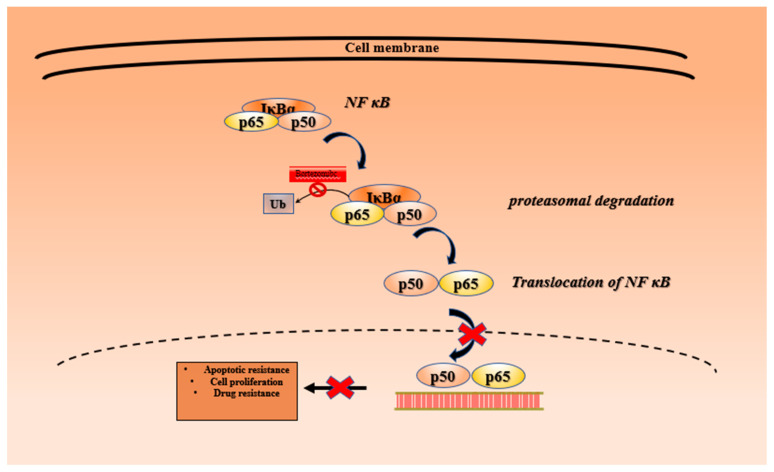
Mechanism of action of bortezomib. **X**: Inhibition.

Bortezomib (BTZ) is a dipeptidyl boronic acid derivative that acts as a reversible proteasome inhibitor primarily adhering to chymotrypsin-like activity of the β5 subunit with a low affinity towards the trypsin-like activity of β1 subunit of the 20S proteasome (Figure 12) [11]. BTZ is the most efficient and successful proteasome inhibitor utilized in clinical practice. The United States Food and Drug Administration (USFDA) has approved BTZ to manage multiple myeloma [182] and mantle cell lymphoma [183]. Various molecular mechanisms are likely involved in the anticancer effect of BTZ. Tremendous preclinical experimentation has been carried out to establish its molecular mechanism and assess its effectiveness alone or in combinational therapy with other chemotherapeutic agents in various malignancies. By blocking the proteasome’s activity, BTZ operates through various molecular mechanisms (Figure 13) to inhibit cancer’s survival pathways, restricts tumor growth, and prevents metastasis and angiogenesis [184]. BTZ reversibly binds to the active site of the 20S subunit and decreases the activity of nuclear factor-kB signaling cascade by inhibiting the proteasome-mediated degradation of inhibitor of NF-kB (IkBs). It confines NF-kB in the cytoplasm by stabilizing its important endogenous inhibitor (IkBs), preventing its nuclear translocation [185]. Following the degradation of IkB, the NF-κB unit translocate into the nucleus and triggers the expression of various genes responsible for apoptotic resistance to cancer cells, cell proliferation, and drug resistance in cancer [15]. BTZ results in cell death in human pancreatic cancer cells by inducing apoptosis mediated through ER stress [186]. Apoptotic mediated death of BTZ has been observed in many cancers, such as gastric cancer [187], lung cancer [188], and ovarian cancer [189].

### 3.2. Carfilzomib

Carfilzomib (CFZ) is a second-generation proteasome inhibitor that addresses some of the shortcomings associated with BTZ. It is an epoxyketone analogue structurally and is chemically distinct from first-generation protease inhibitor BTZ. The CFZ is efficacious as BTZ but displays more specificity towards the chymotrypsin-like site of the proteasome [190]. This drug has been authorized by the FDA (July 2012) to be used as a single agent for the management of multiple myeloma [191]. Proteasome inhibition is more pronounced with CFZ, because it forms an irreversible covalent linkage with the chymotrypsin-like activity of the proteasome. Moreover, because of high specificity and more chemical stability, CFZ results in fewer side-effects than BTZ [192]. Due to its irreversible proteasome inhibition, CFZ delivers a more prolonged and sustained effect in preclinical investigations than BTZ. The most frequent side-effects associated with CFZ are non-hematological, including nausea and fatigue [193].

### 3.3. Ixazomib

Ixazomib (IXZ), a boronic acid derivative, is an orally bioavailable proteasome inhibitor that was FDA approval in 2015 to treat multiple myeloma. It also suppresses the proteasomal activity by binding to the β catalytic subunit, by preferentially blocking the chymotrypsin site [194]. Due to the convenience of oral administration, IXZ has been explored alone or in combination with other chemotherapeutic modalities in various cancers. 

### 3.4. Marizomib (Salinosporamide A)

Marizomib, or salinosporamide A, belongs to the β-lactone-γ-lactam superfamily and is a natural product obtained from marine actinomycete bacteria from the genus Salinispora. It is now being investigated as a potential new orally bioavailable proteasome inhibitor [4]. Marizomib irreversibly binds all the three proteasomal active sites. It has higher affinity towards chymotrypsin-like and trypsin-like sites than the caspase-like site. The irreversible binding characteristics of marizomib are responsible for its increased cytotoxicity and sustained cytotoxicity in tumor cells [195]. Marizomib (NPI-0052) is a second-generation proteasome inhibitor and promising pipeline drug that has demonstrated superior clinical responses in cancer patients resistant to BTZ. Moreover, it has been demonstrated to retain numerous pharmacological characteristics and a wide spectrum of anticancer properties [196,197,198,199]. Marizomib displayed a synergistic effect with BTZ in cytotoxic models of multiple myeloma [197]. Marizomib is well-tolerated and has a propensity to penetrate the CNS (marizomib is associated with adverse effects such as confusion and hallucination), making it a viable treatment for CNS-multiple myeloma. Further research has suggested the role of marizomib as a treatment regimen for brain cancer owing to its CNS penetrating ability [4,200]. 

### 3.5. Oprozomib

Oprozomib (OPZ) is a new orally administered epoxyketone derivative of CFZ. It binds irreversibly to the chymotrypsin-like binding site of proteasome and produces prolonged inhibitory duration [201]. Various clinical trials are ongoing for OPZ in multiple myeloma patients. The chemical modification of CFZ was conceptualized to develop an orally accessible proteasome inhibitor. In preclinical research, apart from the inhibitory effect on the 20S proteasome, OPZ was investigated for its ability to kill cells expressing the P-glycoprotein efflux transporter [4].

### 3.6. Delanzomib

Delanzomib is a boronic acid derivative and proteasome inhibitor that irreversibly inhibits the chymotrypsin and caspase-like activity of the 20S proteasome. Like BTZ, delanzomib downregulates the activity of NF-κB and also induces the induction of apoptosis in cancer cells. More anti-angiogenic and anti-osteoclastic activity were also associated with delanzomib. A synergistic effect was observed when delanzomib was investigated in preclinical research as a combination treatment with dexamethasone and lenalidomide [201]. Table 1 lists all agents inhibiting the proteasome in clinical trials with their chemical and pharmacological characteristics.

## 4. Conclusions

Proteins play an important role in various functions in all human cells and tissues, and the ubiquitin-proteasome system (UPS) governs the renewal of various proteins directly. Cancer, inflammation, neurodegeneration, and other disorders can be triggered by malfunctioning of UPS. Proteasome activity is typically strong in cancer cells, which can break down damaged proteins, resulting in chemotherapeutic treatment resistance. Given the expensive cost of developing new drugs, it is no surprise that natural products have recently become appealing study subjects. Researchers have discovered that phytochemicals are particularly good at controlling the apoptotic mechanisms, mitochondrial stress, and scavenging free radicals associated with cancer. For decades, natural products have been used to cure a wide range of diseases. Though the effects and advantages of these natural products are well-recognized in local communities, they must be officially described through scientific research so that they can be utilized safely in clinical settings as well. Derivatives, prodrugs, and synthetic analogues based on natural products can also be created to improve their safety profiles, avoid bioavailability difficulties, and boost their potency. We evaluated numerous natural products that exhibit proteasome-inhibitory action in this study, presenting different techniques for generating low-cost proteasome-inhibitor anticancer treatment.

## Figures and Tables

**Table 1:** Chemical and pharmacological characteristics of various proteasome inhibitors.

Agent	Active Moiety	Therapeutic (UPS) Target	Binding Kinetics (Towards Proteasome)
Bortezomib	Boronate	CT-L > T-L	Reversible
Carfilzomib	Epoxyketone	CT-L	Irreversible
Ixazomib	Boronate	CT-L	Reversible
Marizomib	β-Lactone	CT-L,T-L > C-L	Irreversible
Oprozomib	Epoxyketone	CT-L	Irreversible
Delanzomib	Boronate	CT-L, C-L	Reversible

CT-L: chymotrypsin-like site, T-L: trypsin-like site, C-L: caspase-like.
